# 5-HT_4_ Receptors Are Not Involved in the Effects of Fluoxetine in the Corticosterone Model of Depression

**DOI:** 10.1021/acschemneuro.1c00158

**Published:** 2021-05-11

**Authors:** Josep Amigo, Emilio Garro-Martinez, Rebeca Vidal Casado, Valerie Compan, Fuencisla Pilar-Cuéllar, Angel Pazos, Alvaro Díaz, Elena Castro

**Affiliations:** † Instituto de Biomedicina y Biotecnología de Cantabria, IBBTEC (Universidad de Cantabria, CSIC, SODERCAN), Departamento de Fisiología y Farmacología, Universidad de Cantabria, 39011 Santander, Spain; ‡ Centro de Investigación Biomédica en Red de Salud Mental (CIBERSAM), Instituto de Salud Carlos III, 28029 Madrid, Spain; § Departamento de Farmacología, Facultad de Medicina, Universidad Complutense, Instituto de Investigación Sanitaria del Hospital Clínico San Carlos (IdISSC), 28040 Madrid, Spain; ∥ Red de Trastornos Adictivos del Instituto de Salud Carlos III, 28029 Madrid, Spain; ⊥ University of Nîmes, Site CARMES, 30000 Nîmes, France

**Keywords:** corticosterone, 5-HT_4_ receptors, knockout mice, fluoxetine, anxiety, depression

## Abstract

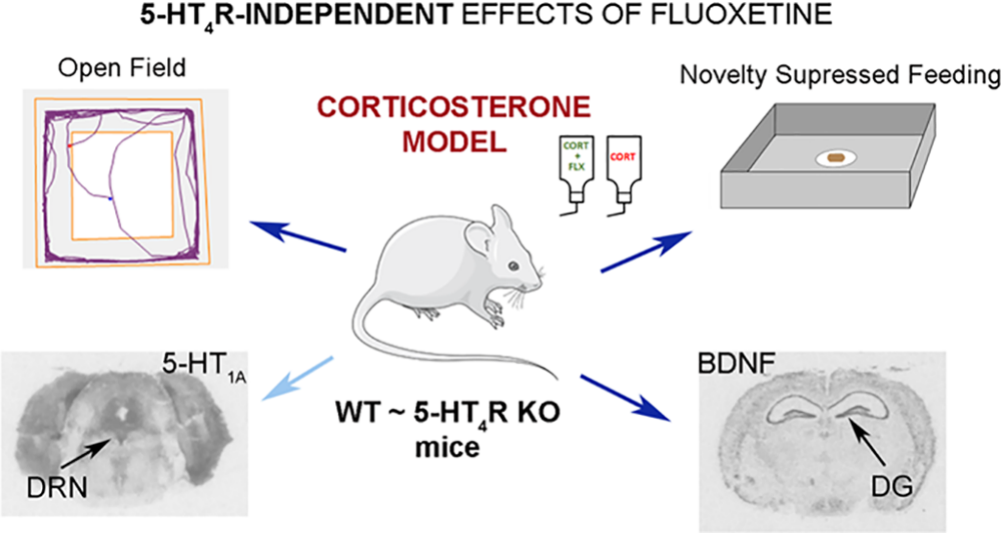

Clinical and preclinical studies report the implication of 5-hydroxytryptamine 4 receptors (5-HT_4_Rs) in depression and anxiety. Here, we tested whether the absence of 5-HT4Rs influences the response to the antidepressant fluoxetine in mice subjected to chronic corticosterone administration, an animal model of depression and anxiety. Therefore, the effects of chronic administration of fluoxetine in corticosterone-treated wild-type (WT) and 5-HT_4_R knockout (KO) mice were evaluated in the open-field and novelty suppressed feeding tests. As 5-HT_1A_ receptor (5-HT_1A_R) and brain-derived neurotrophic factor (BDNF) are critically involved in depression and anxiety, we further evaluated 5-HT_1A_ receptor functionality by [^35^S]GTPγS autoradiography and BDNF mRNA expression by *in situ* hybridization techniques. We found that 5-HT_4_R KO and WT mice displayed anxiety- and depressive-like behavior following chronic administration of corticosterone, as evidenced in the open-field and novelty suppressed feeding tests. In the open-field, a decreased central activity was observed in naïve and corticosterone-treated mice of both genotypes following chronic fluoxetine administration. In the novelty suppressed feeding test, a predictive paradigm of antidepressant activity, chronic treatment with fluoxetine reverted the latency to eat in both genotypes. The antidepressant also potentiated the corticosterone-induced desensitization of the 5-HT_1A_R in the dorsal raphe nucleus. Further, chronic fluoxetine increased BDNF mRNA expression in the dentate gyrus of the hippocampus in corticosterone-treated mice of both genotypes. Therefore, our findings indicate that the behavioral effects of fluoxetine in the corticosterone model of depression and anxiety appear not to be dependent on 5-HT_4_Rs.

## Introduction

Dysfunctions of the serotonin (5-hydroxytryptamine, 5-HT) system in the mammalian brain are related to the pathogenesis of depression.^1^ The serotonin 4 receptors (5-HT_4_Rs) in the medial prefrontal cortex (mPFC) may serve to reduce depressive- and anxiety-like behaviors.^2,3^ The locations of 5-HT4Rs in different structures of the brain are conserved in humans. The highest concentration is found in brain areas implicated in depression- and anxiety-like behaviors, including the limbic system (*e*.*g*. the shell of the nucleus accumbens, the hippocampus), and the lowest in the cerebral cortex.^4−6^ 5-HT_4_Rs commonly exert a positive control of the release of acetylcholine in the frontal cerebral cortex^7^ and 5-HT in the dorsal raphe nucleus (DRN^3^). The DRN is the main origin of serotonergic neurons in the forebrain. 5-HT_4_Rs serve to enhance the activity of DRN 5-HT neurons, not from the DRN (they are apparently absent) but from the ventral mPFC.^2,8,9^

Studies in humans, using positron emission tomography, reveal the relationship between depression and low levels of 5-HT_4_Rs in the caudate-putamen.^10^ Analyses in brain samples from individuals who committed suicide revealed higher concentrations in both 5-HT_4_Rs and cyclic adenosine monophosphate (cAMP) in the frontal cerebral cortex and the caudate-putamen than those in controls.^11^ Accordingly, local stimulation of 5-HT_4_Rs in the nucleus accumbens (NAc) induced an increased activity of rewarding signaling (cAMP/PKA: protein kinase A/CART: cocaine- and amphetamine-regulated transcript) in freely moving mice,^12^ in agreement with the positive coupling of 5-HT_4_Rs with adenylate cyclase, as previously seen in neurons *in vitro*.^13^ Preclinical studies also relate less activity of 5-HT4Rs with depressive- and anxiety-like behaviors.^2,8,9,14−16^

5-HT_4_R knockout (KO) mice displayed anxiety-like behavior in response to stress and novelty,^14^ showed less motor reactivity to novelty,^14,17,18^ and exhibited anhedonia^16^ and long-term memory deficits.^17^ These mutant mice also exhibited abnormal feeding response, *i*.*e*. attenuated hypophagia (reduced food intake) following unexpected restraint stress.^14^ Adult restoration of 5-HT_4_Rs expression in the mPFC (genic therapy) rescues hypophagia and specific molecular changes related to depression resistance in the DRN [5-HT release elevation, 5-HT_1A_ receptor (5-HT_1A_R), and 5-HT transporter reductions] in stressed 5-HT_4_R KO mice.^3^ The levels of 5-HT4Rs were also reduced in the dorsal and ventral hippocampus in the Flinders-sensitive line rat model of depression.^19^ Increases in the levels of 5-HT4Rs in the ventral hippocampus and the striatum were reported in other animal models of depression, including olfactory bulbectomized (OB) and glucocorticoid heterozygous receptor mice, suggesting context-dependent implications of 5-HT_4_Rs.^20^

The 5-HT4Rs are also implicated in the molecular mechanisms of action of antidepressants, and 5-HT4R compounds could serve as antidepressants.^9,15,21,22^ The desensitization of 5-HT_4_Rs is however observed in the striatum and the hippocampus of rats chronically treated with classic antidepressants like fluoxetine^23^ and venlafaxine.^24^ Predictive behavioral paradigms indicate that the activation of 5-HT4Rs could contribute to anxiolytic and antidepressant effects of the selective 5-HT reuptake inhibitor (SSRI) fluoxetine.^16,25^ In addition, 5-HT_4_Rs may serve to the neurogenic effects of fluoxetine in both naïve^26^ and corticosterone-treated mice.^25^

Following up these studies, here, we further explored behavioral, neurochemical, and molecular consequences of *Htr*4 gene mutation leading to the absence of 5-HT_4_Rs. Naïve 5-HT_4_R KO mice displayed reduced (−50%) firing activity of the DRN 5-HT neurons associated with diminished tissue levels of 5-HT and the main metabolite, 5-hydroxy indole acetic acid.^9^ Other changes in the DRN of 5-HT_4_R KO mice included increases in the levels of 5-HT transporter sites and mRNA and a decrease in the density of 5-HT_1A_R (as in the dorsal hippocampus and the septum) without any changes in the mRNA levels of 5-HT_1A_R.^3,9^ Naïve 5-HT_4_R KO mice also exhibited an alteration in the levels of critical markers related to stress and depression, as brain-derived neurotrophic factor (BDNF), Arc and trkB in the cortical and limbic structures in the brain.^16^ In the present study, we first tested whether the absence of 5-HT4Rs modifies the behavioral responses induced by the antidepressant fluoxetine in mice subjected to the corticosterone model, classically used to mimic anxiety and depression in humans, and to evaluate the antidepressant/anxiolytic effects of drugs. We then assessed the adaptive changes in the functionality of 5-HT1AR in *ex vivo* samples using the [^35^S]GTPγS autoradiography technique and the mRNA levels of BDNF by *in situ* hybridization.

## Results and Discussion

### Corticosterone Model in WT and 5-HT_4_R KO Mice: Effect of Fluoxetine

**Figure 1:**
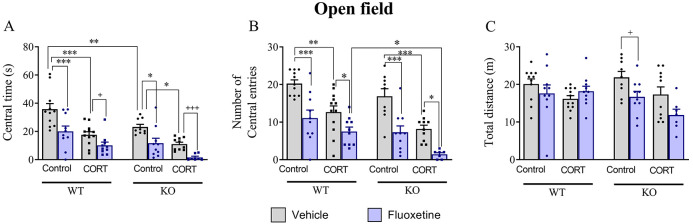
Behavioral effects following chronic fluoxetine treatment in control and corticosterone-treated mice in the OF test. Central time (A), number of central entries (B), total distance traveled (C). Three-way ANOVA analysis showed an effect of genotype and treatment in all OF parameters, an effect of the model in central activity, and a significant model × treatment interaction on total distance (Table S1, supplementary statistical report). Data are mean ± SEM: *p < 0.05, **p < 0.01, and ***p < 0.001 (Newman–Keuls *post hoc* test); ^+^*p* < 0.05; ^+++^*p* < 0.001 (Student’s unpaired *t*-test).

WT and 5-HT_4_R KO mice exhibited an enhanced anxiety- and depressive-like behavior following chronic administration of corticosterone as evidenced in the open-field (OF) and novelty suppressed feeding (NSF) tests. In the OF, WT-CORT mice spent less time and entered less in the center (Figures 1A, B) and presented a similar total distance traveled (Figure 1C) compared with WT-control group. Similarly, 5-HT_4_ KO-CORT mice showed a decrease in central time and central entries together with no significant changes in the total distance traveled (Figures 1A–C). Then, we assessed the effect of chronic administration of fluoxetine in mice of both genotypes in the OF (Figures 1A–C). In corticosterone-treated WT mice, 14-day treatment with fluoxetine significantly reduced OF central time (42.5%) and entries (45.1%). In corticosterone-treated 5-HT_4_R KO mice, fluoxetine also significantly reduced OF central time (85.7%) and entries (82.6%). Chronic fluoxetine induced a similar reduction (around 45–50%) of both OF central parameters in the control groups of WT and KO mice (Figures 1A and B).

**Figure 2:**
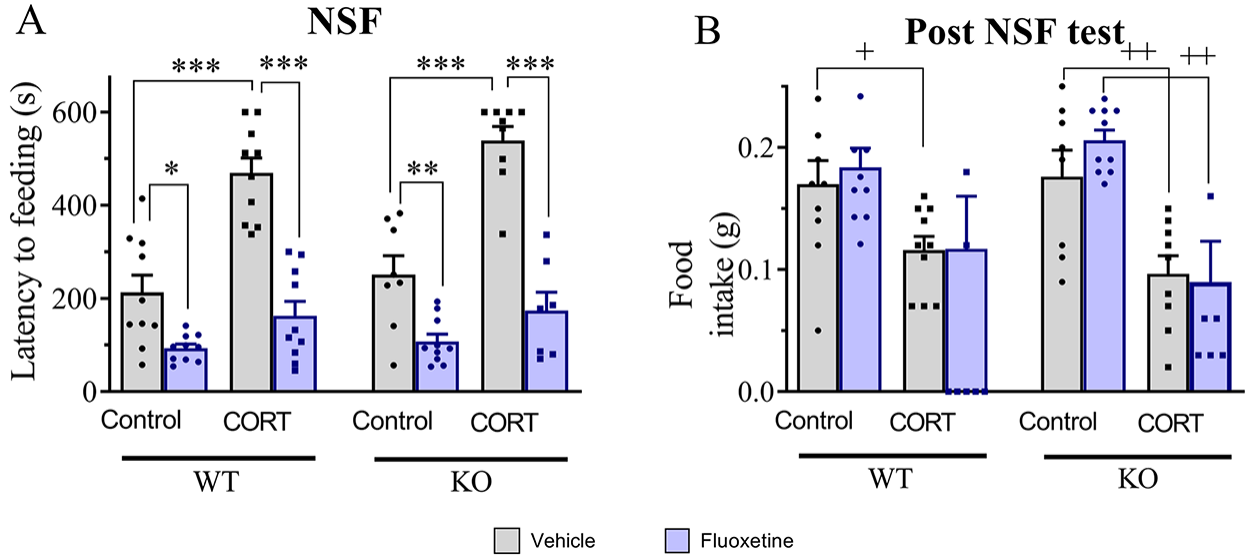
Behavioral effects of chronic fluoxetine treatment in control and corticosterone-treated mice in the NSF test. Latency to feeding (A) and post-NSF food intake (B). Three-way ANOVA analysis showed an effect of genotype in both parameters, an effect of treatment in the latency to feeding, and a significant genotype × treatment interaction in the latency to feeding (Table S1, supplementary statistical report). Data are mean ± SEM: *p < 0.05, **p < 0.01, and ***p < 0.001 (Newman–Keuls *post hoc* test); ^+^*p* < 0.05; ^+2^*p* < 0.01 (Student’s unpaired *t*-test).

Next, we evaluated the behavior of corticosterone-treated WT and KO mice, and the effect of chronic fluoxetine, in the NSF which assesses context-dependent anxiety and a predictive paradigm used to evaluate the effect of antidepressant treatments. WT-CORT mice needed more time for eating (+120% vs WT-control; Figure 2A). Similarly, corticosterone-treated KO mice showed an increased latency to eat (+114.5% vs KO-control mice; Figure 2A). Chronic fluoxetine significantly reduced the latency to eat in both corticosterone-treated genotypes (WT-CORT-flx: 85.7% and KO-CORT-flx: 82.6%; Figure 2A.). Moreover, fluoxetine also decreased the latency to feeding in the control groups of both genotypes (around 50%) (Figure 2A).

Regarding the amount of food intake measured in the 5 min session performed in their home cages immediately after the NSF test, both WT-CORT and KO-CORT showed a reduced food intake (Figure 2B). Chronic fluoxetine had no effect in food intake in control and corticosterone-treated mice of both genotypes (Figure 2B).

It is well-known that SSRIs can be effective in treating anxiety and depression in humans but, in some cases, antidepressants can favor anxiety. The mechanisms involved in this side effect of antidepressants remain unclear. Here, we show that the chronic treatment with fluoxetine induced an anxiogenic-like effect (reduced central activity) in control and corticosterone-treated mice of both genotypes in the open-field test. The 5-HT_4_R KO mice exhibit a hyperanxiety-like behavior under basal conditions,^14,16^ which may explain their apparent higher “anxiogenic score” under the present fluoxetine/corticosterone-treatment conditions. The anxiogenic effect of fluoxetine in mice has been earlier reported, not only in the open-field but also in the elevated plus maze^27^ and in rats in the hole-board test.^28^ An anxiogenic response of juvenile mice to fluoxetine was also reported independently of the strains and tests used.^29^ In contrast to our finding, an anxiolytic effect of fluoxetine was reported in corticosterone-treated mice in the open-field test.^25,30^ This discrepancy may be due to the different duration of the treatment (4 weeks versus 2 weeks in the present study), and/or different strains of mouse (C57BL/6 versus 129SvTer in the present study), and age (between 4 to 8 weeks versus 12 in the present study). We previously reported no changes in anxiety-like parameters following chronic fluoxetine treatment in bulbectomized WT and 5-HT_4_R KO mice using the OF test,^16^ suggesting additional model-dependent differences. All of these preclinical findings introduce the need for additional investigations as duration and initial condition of antidepressant treatments can, in some patients, trigger or enhance anxiety- and panic-like responses.^31,32^

The novelty suppressed feeding test is widely used to assess not only the acute effects of anxiolytics but also as a predictive paradigm of chronic antidepressants.^33^ In both genotypes, chronic fluoxetine was effective in control and corticosterone-treated groups. In contrast with the present findings, GR125487, a 5-HT_4_R antagonist, is reported to prevent the anxiolytic/antidepressant effect of fluoxetine in the corticosterone animal model.^25^ It is worth noting that GR125487 binds also to 5-HT_3_ receptors,^34^ and some studies report that the blockade of these receptors induces anxiolytic effects in mice.^35,36^ In addition, we must consider that a pharmacological antagonism must not parallel the genetic deletion because adaptive mechanisms in the serotonergic system in 5-HT_4_R KO mice may be present. In this sense, 5-HT_4_R KO mice display hyperanxiety-like behavior^14^ consistent with a reduced density of 5-HT_1A_R in the dorsal hippocampus.^9^ 5-HT_4_R KO mice also display increased levels of 5-HT transporter in the DRN.^3,9^ Therefore, the anxiety-related phenotype of 5-HT_4_R KO mice may then likely result from these cumulative adaptations in the serotonergic system.

### *In Vitro* 5-HT_1A_R Functionality in Corticosterone-Treated WT and 5-HT_4_R KO Mice: Effect of Fluoxetine

**Figure 3:**
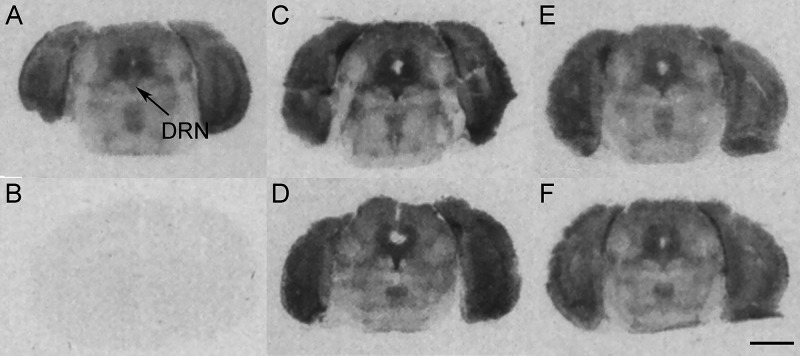
Representative autoradiograms of [^35^S]GTPγS binding. (A) Basal binding and (B) nonspecific binding. 8-OH-DPAT-induced stimulation of [^35^S]GTPγS binding in (C) WT-control-vehicle, (D) WT-control-fluoxetine, (E) WT-CORT-vehicle, and (F) WT-CORT-fluoxetine. DRN: dorsal raphe nucleus. Scale bar = 2 mm.

Following chronic exposure to corticosterone, a reduction in [^35^S]GTPγS binding induced by 8-OH-DPAT was observed at the level of the DRN in WT-CORT mice but not in KO-CORT mice. Chronic treatment with fluoxetine potentiated the 5-HT_1A_ autoreceptor desensitization observed in WT-CORT mice but had no effect in KO-CORT mice. In KO-control mice, 8-OH-DPAT-induced [^35^S]GTPγS binding was decreased in the DRN when compared with WT-control mice as reported by Amigó et al.^16^ Furthermore, chronic fluoxetine had no effect in WT- and KO-control mice (Table 1, Figure 3 and Table S2, supplementary statistical report).

**Table 1:** Effect of chronic fluoxetine on the stimulation of [^35^S|GTPγS binding induced by 8-OH-DPAT in control and corticosterone-treated mice^a^

WT					KO			
	control-VH	control-Flx	CORT-VH	CORT-Flx	control-VH	control-Flx	CORT-VH	CORT-Flx
DRN	44.3 ± 3.1	40.5 ± 5.4	28.5 ± 4.3*	11.5 ± 4.5^+^	26.9 ± 4.8^##^	37.5 ± 11.2	30.3 ± 3.5	20.0 ± 6.7
CA1	129.1 ± 12.0	79.7 ± 16.2^+^	143.7 ± 9.9	96.4 ± 8.1^+^	123.6 ± 9.2	46.6 ± 11.5^+++^	118.1 ± 11.7	101.4 ± 12.0
CA3	29.6 ± 9.4	17.1 ± 4.3	29.5 ± 6.1	14.1 ± 7.0	14.1 ± 7.0	18.4 ± 11.7	21.2 ± 8.0	32.6 ± 10.6
DG	50.7 ± 7.0	17.9 ± 6.0^+^	59.6 ± 10.7	51.5 ± 8.7	40.4 ± 2.2	1.1 ± 10.0^++^	36.5 ± 8.3	74.9 ± 10.8^+^

a DRN: dorsal raphe nucleus, CA1, CA3: CA1, CA3 fields of the hippocampus; DG: dentate gyrus of the hippocampus. Data are mean ± SEM of *n* = 6–8 mice per group. Values are expressed as percentage of 8-OH-DPAT stimulated [^35^S]GTPγS binding. *p < 0.05 vs control-VH group; ^+^*p* < 0.05, ^++^*p* < 0.01, ^+++^*p* < 0.001, effect of fluoxetine vs respective VH-treated groups; ^##^*p* < 0.01 vs WT-control-VH group (Newman–Keuls *post hoc* test).

In the hippocampus, chronic administration of corticosterone did not modify 8-OH-DPAT-induced stimulation of [^35^S]GPTγS binding in any field in mice of both genotypes, while different changes were detected following chronic fluoxetine treatment depending on the genotype and the brain area examined (Table 1 and Table S2, supplementary statistical report). In the CA1 field, a decrease in 8-OH-DPAT-induced stimulation of [^35^S]GPTγS binding was observed in WT-CORT, but not in KO-CORT mice, following fluoxetine treatment. In the DG, an increase in 8-OH-DPAT-induced stimulation of [^35^S]GPTγS binding was observed in fluoxetine-treated KO-CORT but not in WT-CORT mice counterparts. However, a similar desensitization was observed in the CA1 field and DG in both WT-control and KO-control mice following fluoxetine treatment. Finally, in the CA3 field, no changes were observed in any experimental conditions (Table 1 and Table S2, supplementary statistical report).

Changes in 5-HT_1A_R expression and functionality have been linked to anxiety- and depressive-like states,^37,38^ the anxiolytic/antidepressant effects^39,40^ and the vulnerability or resilience to stress-related disorders.^41,42^ In our study, we detected desensitization of 5-HT_1A_R in the DRN in WT mice following chronic corticosterone administration, consistently with earlier studies,^43,44^ and other observations in animal studies using different stressful conditions (chronic unpredictable stress,^45^ maternal deprivation,^46^ and social defeat^47^). Accordingly, 5-HT_1A_R density is also reduced in the midbrain following suicide^48^ and in the DRN of humans with depression,^49−51^ though increases have also been reported in human studies.^52−54^ Corticosterone-induced 5-HT_1A_R desensitization was potentiated by chronic administration of fluoxetine in corticosterone-treated-WT mice, an adaptive change that could contribute to the behavioral effect of fluoxetine in the novelty suppressed feeding, a predictive paradigm of antidepressant activity. It has been reported that chronic SSRI treatments are associated with 5-HT_1A_ autoreceptor desensitization^55^ and, conversely, high expression and functionality of DRN 5-HT_1A_R is associated with a low efficacy of antidepressants.^56,57^ By contrast, no desensitization of the 5-HT_1A_R in the DRN in 5-HT_4_R KO mice was observed following chronic administration of corticosterone alone or in combination with fluoxetine. However, it is worth mentioning that 5-HT_4_R KO mice already exhibit a lower density^9^ and functionality (^16^ and present results) of DRN 5-HT_1A_R which may account for the lack of further downregulation of these receptors.

In relation with the hippocampal 5-HT_1A_R, chronic corticosterone administration did not alter their functionality in WT and KO mice, in line with a previous study,^43^ although a desensitization was reported in another animal model, the olfactory bulbectomy in mice, in CA1-CA2 hippocampal areas.^58^ Also, our study shows that fluoxetine induced desensitization of hippocampal 5-HT_1A_R (CA1 and DG) in control animals of both genotypes. The regulation of these 5-HT_1A_R by antidepressants is quite controversial. In rodent studies, long-term SSRI treatment induced an increase^39,59,60^ or no change,^61,62^ and human studies have not drawn conclusive findings.^53,63,64^ In our study, chronic fluoxetine induced an increase in the functionality of 5-HT_1A_R in the DG in corticosterone-treated KO mice (an opposite finding to that observed in naïve counterparts). Therefore, we must be cautious when interpreting the regulation of hippocampal 5-HT_1A_R by antidepressants because the findings may be not the same, or even opposite, in naïve or animals subjected to a model of depression.

### mRNA Levels of BDNF in Corticosterone-Treated Animals: Effect of Fluoxetine

**Figure 4:**
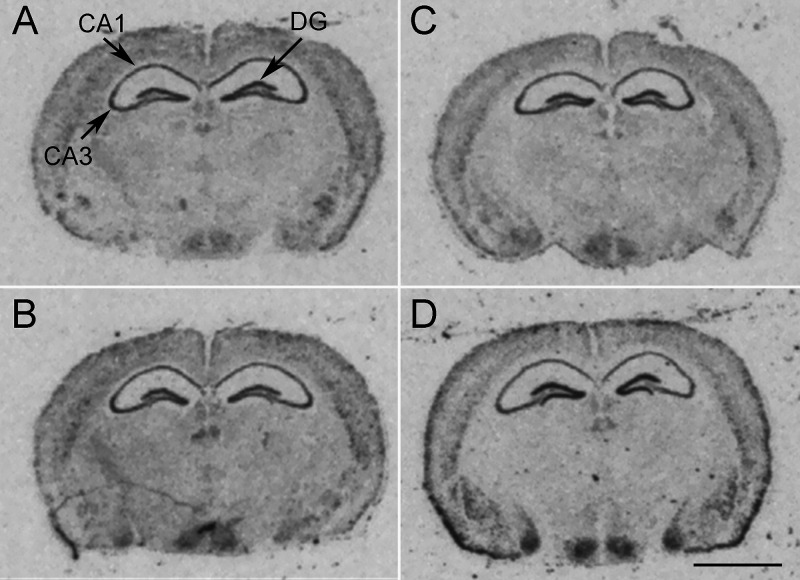
Representative autoradiograms of BDNF mRNA expression. (A) WT-control-vehicle; (B) WT-control-fluoxetine; (C) WT-CORT-vehicle; and (D) WT-CORT-fluoxetine. CA1 and CA3: CA1 and CA3 fields of the dorsal hippocampus and DG: dentate gyrus. Scale bar = 2 mm.

The mRNA levels of BDNF in the dorsal hippocampus (CA1, CA3, and DG) of corticosterone-treated mice of both genotypes were similar to those observed in controls counterparts (Table 2 and Table S2, supplementary statistical report). Chronic treatment with fluoxetine increased the mRNA levels of BDNF in the DG, but not in CA1 and CA3 fields, in corticosterone-treated mice of both genotypes (WT: +44.0% and 5-HT_4_R KO: +55.5% vs the respective corticosterone-vehicle group). Finally, the antidepressant did not modify the hippocampal mRNA levels of BDNF in control mice of both genotypes (Table 2, Figure 4 and Table S2, supplementary statistical report).

**Table 2:** Effect of Chronic Fluoxetine on the Hippocampal mRNA Levels of BDNF in Control and Corticosterone-Treated Mice^a^

WT					KO			
	control-VH	control-Flx	CORT-VH	CORT-Flx	control-VH	control-Flx	CORT-VH	CORT-Flx
CA1	18.8 ± 1.2	19.9 ± 0.9	20.2 ± 1.3	18.4 ± 2.0	15.8 ± 1.2	14.3 ± 0.8	14.4 ± 0.8	12.1 ± 1.4
CA3	31.0 ± 3.5	30.2 ± 2.0	29.1 ± 2.4	33.0 ± 5.3	30.8 ± 4.3	26.6 ± 2.6	27.4 ± 2.6	25.5 ± 4.2
DG	41.4 ± 3.2	19.0 ± 3.6	47.0 ± 2.4	67.7 ± 5.7^++^	40.5 ± 2.6	27.5 ± 3.6^+^	39.3 ± 2.5	65.1 ± 8.4^+^

a CA1: CA1 field of the hippocampus, CA3: CA3 field of the hippocampus; DG: dentate gyrus of the hippocampus. Data are mean ± SEM of *n* = 6–9 mice per group, expressed in nCi/g tissue equivalent. ^++^*p* < 0.01 *vs* respective vehicle-treated group (Newman–Keuls *post hoc* test).

The study of the implication of BDNF in depression and the effects of different antidepressants has been largely investigated.^65,66^ Administration of BDNF^67,68^ and overexpression of BDNF in the hippocampus^69^ induces antidepressant effects, while BDNF knockdown in the DG of rats produces depressive-like behavior.^70^ In our study, chronic corticosterone treatment had no effect on the mRNA levels of BDNF in mice of both genotypes. Reduced mRNA and protein levels of BDNF were reported in the whole hippocampus in mice using other techniques such as RT-PCR and ELISA.^71,72^ These differences are commonly reported between studies when *in situ* hybridization and biochemical techniques are used. The *in situ* hybridization technique provides an anatomical local resolution, while the other techniques detect “a global change” in the whole sample tissue. Finally, changes in the levels of protein, including BDNF, do not always parallel those of mRNA.

Chronic fluoxetine increased mRNA levels of BDNF in the DG in corticosterone-treated mice of both genotypes, a finding that may be associated with the different behavioral effects of fluoxetine in the OF versus novelty suppressed feeding test.^73^ For instance, the overexpression of BDNF in the hippocampal astrocytes produces an antidepressant effect in the novelty suppressed feeding test.^74^ Therefore, this upregulation in hippocampal BDNF mRNA levels, together with the desensitization of DRN 5-HT_1A_R, may contribute to the antidepressant effect of fluoxetine as evidenced in the novelty suppressed feeding test. However, changes in the levels of BDNF (mRNA or protein) exert opposite effects in anxiety-like behaviors because its overexpression in the hippocampus induces anxiogenic-like behavior in the OF^69^ and the light/dark box test,^75^ but an anxiolytic effect in the elevated plus maze.^76^ This 5-HT_4_R-independent effect of fluoxetine highlights the implication of hippocampal BDNF in the anxiolytic/antidepressant actions of this SSRI under pathological conditions.

In conclusion, the present study excludes an outstanding role of 5-HT_4_Rs in the corticosterone model of depression because 5-HT_4_R KO mice present behavioral manifestations similar to those of WT mice. Furthermore, chronic treatment with fluoxetine exerts 5-HT_4_R-independent effects in depression- and anxiety-related behaviors. Finally, the behavioral effects of fluoxetine in this animal model of depression are associated with the regulation of 5-HT_1A_R functionality and hippocampal BDNF expression.

## Material and Methods

### Animals

The 5-HT4R KO and WT male mice (3 months old, 25 ± 1 g) were obtained from the breeding of 129SvTer 5-HT4R heterozygote mice.^14^ They were housed (*n* = 4–5 per cage) in the animal house of the University of Cantabria in a temperature-controlled environment with 12 h light/dark cycle, with food and water available *ad libitum*. All experiments were carried out with the approval of the Animal Care Committee of the Universidad de Cantabria and were performed following Spanish legislation (Real Decreto 53/2013) and the European Communities Council Directive 2010/63/UE on “Protection of Animals Used in Experimental and Other Scientific Purposes”.

### Drugs and Chemicals

[^35^S]-2′-deoxyadenosine-5′-(α-thio)triphosphate (dATP) and [^35^S]-guanosine-5′-(γ-thio)triphosphate (GTPγS) were used at a specific activity of 1250 Ci/mmol (PerkinElmer). Fluoxetine hydrochloride and (±)-8-hydroxy-2-dipropylaminotetralin hydrobromide (8-OH-DPAT) were purchased from Tocris Bioscience, and corticosterone hemisuccinate (4-pregnen-11b-DIOL-3 20-DIONE 21-hemisuccinate) was from Steraloids. All other chemicals used were of analytical grade.

### Corticosterone Model of Depression and Anxiety and Pharmacological Treatments

**Figure 5:**
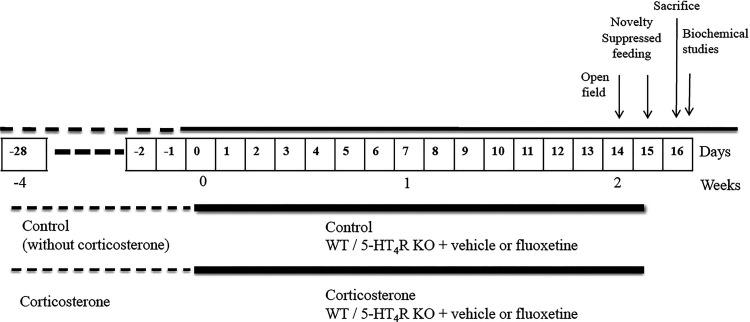
Experimental design. Chronic administration of fluoxetine in control (without corticosterone) and corticosterone-treated WT and 5-HT_4_R KO mice. Biochemical analyses: [^35^S]GTPγS autoradiography of 5-HT_1A_R and BDNF *in situ* hybridization.

To induce the corticosterone model of depression, WT and 5-HT4R KO mice were administered corticosterone in their drinking water (45 mg/L of corticosterone hemisuccinate) for four weeks (Figure 5), as reported.^77^ Corticosterone solutions were stocked in opaque bottles and were replaced every seven days to avoid degradation, and the volume of the consumed solution was evaluated every three-day period to adjust concentrations when necessary. Mice of both genotypes consumed an identical volume of corticosterone solution along with the treatment (data not shown). Following four weeks of treatment, behavioral analyses were conducted to confirm corticosterone-induced depressive- and anxiety-like behavior before the initiation of fluoxetine treatment.

### Pharmacological Treatments and Experimental Groups

In mice of both genotypes, the effects of chronic administration of fluoxetine (160 mg/L, equivalent to 25 mg/kg/day) or its vehicle (drinking water) were evaluated in control (without corticosterone) and corticosterone-treated mice (Figure 5). The volume of the consumed solution of fluoxetine was evaluated every three-day period to adjust concentrations when necessary. At the end of the behavioral assessment, mice were sacrificed, and their brains were extracted and stored at −80 °C until used for *ex vivo* studies ([^35^S]GT PγS 5-HT_1A_R and *in situ* hybridization of mRNA encoding BDNF).

### Behavioral Studies

Behavioral assessment was carried out following 14 days of treatment with fluoxetine and 24 h following the last administration, as described.^58^ Behavioral tests were conducted during the light phase (9:00 a.m. to 5:00 p.m.) beginning by the least stressful test (OF) and followed by the most stressful test (NSF), carried out during two different consecutive days for minimizing potential side effects.

The OF test was conducted for evaluating motor reactivity to novelty, and anxiety-like behavior, as previously utilized.^16^ The OF environment was a wooden square chamber placed in a wooden box (50 cm × 50 × 30 cm) with the center of the arena highly illuminated (400 lx). Mice were placed in a corner of the OF at the beginning of the test. Mice behavior was automatically video-tracked for 5 min, and behavioral parameters (time spent and the number of entries in the center, and the total traveled path length) were recorded using the Any-maze software (Stoelting Co., United States).

The NSF test was employed as reported previously.^16^ The NSF was conducted following a period of 24-h of total food deprivation. The following day, each mouse was placed in a corner of the box (50 cm × 50 × 30 cm) with wood chip bedding and a food pellet (±2 g) placed in the center (40–50 lx). The time latency (expressed in seconds) to eat the pellet was automatically recorded for 10 min maximum using the Any-maze software (Stoelting Co., United States). At the end of the test, mice were placed back into their home cage to evaluate the amount of food eaten during a 5 min session immediately after the NSF test.

### Autoradiography Study of 5-HT_1A_R-Dependent Stimulation of [^35^S]GTPγS Binding

Mice were sacrificed 24 h following the last behavioral test, *i*.*e*. the NSF, and their brains were rapidly removed and frozen immediately on dry ice and then stored at −80 °C until sectioning. Coronal brain 14 μm thick sections were cut at −20 °C using a microtome cryostat, thaw-mounted in slices, and stored at −20 °C until used for [^35^S]GTPγS binding assays. Labeling of brain sections with [^35^S]GTPγS was carried out, as previously described,^78^ to evaluate the functionality of 5-HT_1A_R using the agonist 8-OH-DPAT (10 μM). Slide-mounted sections were preincubated for 30 min at room temperature in a buffer containing 50 mM Tris-HCl, 0.2 mM EGTA, 3 mM MgCl_2_, 100 mM NaCl, 1 mM dithiothreitol, and 2 mM GDP at pH 7.7. Slides were then incubated for 2 h in the same buffer containing adenosine deaminase (3 mU/mL) with [^35^S]GTPγS (0.04 nM), and successive brain sections were coincubated with 8-OH-DPAT (10 μM). The nonspecific binding was determined in the presence of 10 μM GTPγS. After the incubation, the brain sections were washed twice for 15 min in cold 50 mM Tris-HCl buffer (pH 7.4), rinsed in cold distilled water, and then dried under a cold air stream. Sections were exposed to film BioMax MR (Carestream) together with [^14^C] microscales at 4 °C for 2 days. The autoradiograms generated were analyzed and quantified using a computerized image analysis Scion Image software (Scion Corporation, MD, United States). The data from the [^35^S]GTPγS autoradiography of 5-HT_1A_R were represented as the percentage of stimulation of [^35^S]GTPγS binding induced by 8-OH-DPAT. This parameter was calculated as a percentage 8-OH-DPAT-stimulated binding compared with the specific basal binding.

### BDNF *in Situ* Hybridization

Coronal brain 14 μm-thick sections were collected as described above. As adapted from ref (79), we utilized oligonucleotide complementary sequence to mRNA sequence encoding BDNF 5′-GGTCTCGTAGAAATATTGGTTCAGTTGGCCTTTTGATACCGGGAC-3′,^80^ which was 3′ end-labeled with [^35^S]dATP using terminal deoxynucleotide transferase and added 250 000 c.p.m./slide, with hybridization buffer (50% deionized formamide, 4× standard saline sodium citrate (SSC), sodium phosphate 10 mM pH 7.0, sodium pyrophosphate 1 mM, 10% dextran sulfate, 5× Denhardt’s solution, 200 μg/mL salmon sperm DNA, 100 μg/mL poly-A, heparin 0.12 mg/mL, and 20 mM dithiothreitol. Following incubation at 42 °C for 16 h, brain sections were washed at 50 °C in 2× SSC buffer with DTT 1 M twice for 30 min followed by 3 washes of 5 min at room temperature with 1× SSC, 0.1× SSC, and ethanol 80% successively. Finally, brain sections on slides were washed in ethanol 96% for 1 min at room temperature. Sections were then air-dried and exposed to BioMax MR films (Carestream) together with [^14^C] microscales at −20 °C for 3 weeks. The control of specificity was performed using the nonlabeled probe (at a concentration 1000 times higher). Optical density values were calibrated using [^14^C] microscales using a computerized image analysis Scion Image software (Scion Corporation, MD, United States). The autoradiograms were analyzed and quantified using a computerized image analysis from Scion Image software (Scion Corporation, MD, United States). The data were expressed in nCi/g of estimated tissue equivalent.

### Statistical Analyses

Three-way ANOVA, followed by Newman–Keuls *post hoc* tests, Student’s *t* test, or linear regression were performed when it was appropriate (see Supporting Information for detailed statistical analyses). The level of significance was set at *p* < 0.05. Graph editing and statistical analyses were performed using the GraphPad Prism Software version 8.2 (GraphPad, San Diego, CA, United States).

## Supporting Information Available

Statistical analysis report for behavioral and biochemical studies (PDF)
