# Supplementary material for: 5-HT4 Receptors Are Not Involved in the Effects of Fluoxetine in the Corticosterone Model of Depression
Source: ACS Chem Neurosci. 2021 May 11;12(11):2036–44. doi: 10.1021/acschemneuro.1c00158 (PMC8459452; doi:10.1021/acschemneuro.1c00158)
Supplement: Supplementary file 1 — cn1c00158_si_001.pdf [file cn1c00158_si_001.pdf]

5-HT_4 Receptors Are Not Involved in the Effects of Fluoxetine in the Corticosterone Model of Depression

Authors

Josep Amigo[a,b], Emilio Garro-Martinez[a,b,1], Rebeca Vidal Casado[c,d], Valerie Compan[e], Fuencisla Pilar-Cuéllar[a,b,], Angel Pazos[a,b], Alvaro Díaz[a,b,*], Elena Castro[a,b,*]

Affilations:

[a]Instituto de Biomedicina y Biotecnología de Cantabria, IBBTEC (Universidad de Cantabria, CSIC, SODERCAN), Departamento de Fisiología y Farmacología, Universidad de Cantabria, 39011 Santander, Spain
[b]Centro de Investigación Biomédica en Red de Salud Mental (CIBERSAM), Instituto de Salud Carlos III, Spain
[c]Departamento de Farmacología, Facultad de Medicina, Universidad Complutense, Instituto de Investigación Sanitaria del Hospital Clínico San Carlos (IdISSC), Spain
[d]Red de Trastornos Adictivos del Instituto de Salud Carlos III, Madrid, Spain
[e]University of Nîmes, Site CARMES, 30 000 Nîmes, France

[1]Present address: Department of Integrative Medical Biology (IMB). Umeå universitet, 901 87 Umeå, Sweden.

SUPPLEMENTARY INFORMATION

Table S1. Statistical analysis report (Behavioural studies)

Measurement	Three-way ANOVA	F(DFn, DFd)	*p*
Central time OF (Fig 1A)	Genotype	F (1,71) = 37.90	< 0.001
	Model	F (1,71) = 19.55	< 0.001
	Treatment	F (1,71) = 29.29	< 0.001
	Genotype x model	F (1,71) = 0.4973	0.4830
	Genotype x treatment	F (1,71) = 1.601	0.2098
	Model x treatment	F (1,71) = 0.07886	0.7797
	Genotype x model x treatment	F (1,71) = 0.5802	0.4488
Central entries OF (Fig 1B)	Genotype	F (1,71) = 34.95	< 0.001
	Model	F (1,71) = 16.53	< 0.001
	Treatment	F (1,71) = 49.61	< 0.001
	Genotype x model	F (1,71) = 0.5923	0.4441
	Genotype x treatment	F (1,71) = 2.451	0.1219
	Model x treatment	F (1,71) = 0.2150	0.6443
	Genotype x model x treatment	F (1,71) = 0.07437	0.7859
Total distance OF (Fig 1C)	Genotype	F (1,71) = 7.905	< 0.01
	Model	F (1,71) = 0.9031	0.3452
	Treatment	F (1,71) = 5.974	< 0.05
	Genotype x model	F (1,71) = 1.812	0.1825
	Genotype x treatment	F (1,71) = 0.8859	0.3498
	Model x treatment	F (1,71) = 5.029	< 0.05
	Genotype x model x treatment	F (1,71) = 1.09	0.2958
NSF: Latency to feeding (Fig 2A)	Genotype	F (1,66) = 66.33	< 0.001
	Model	F (1,66) = 2.622	0.1102
	Treatment	F (1,66) = 121.9	< 0.001
	Genotype x model	F (1,66) = 0.08871	0.7668
	Genotype x treatment	F (1,66) = 22.67	< 0.001
	Model x treatment	F (1,66) = 1.027	0.3146
	Genotype x model x treatment	F (1,66) = 0.1357	0.7138
NSF: post-test (Fig 2B)	Genotype	F (1,66) = 22.86	< 0.001
	Model	F (1,66) = 0.07228	0.7889
	Treatment	F (1,66) = 0.3263	0.5698
	Genotype x model	F (1,66) = 1.282	0.2617
	Genotype x treatment	F (1,66) = 0.5513	0.4604
	Model x treatment	F (1,66) = 0.01606	0.8995
	Genotype x model x treatment	F (1,66) = 0.1285	0.7211

Table S2. Statistical analysis report (Biochemical studies)

Measurement	Three-way ANOVA	F(DFn, DFd)	*p*
[^{35}S]GTPγS Binding: DRN (Table 1)	Genotype	F (1,49) = 14.92	< 0.001
	Model	F (1,49) = 0.09444	0.7599
	Treatment	F (1,49) = 1.134	0.2922
	Genotype x model	F (1,49) = 7.159	< 0.05
	Genotype x treatment	F (1,49) = 4.245	< 0.05
	Model x treatment	F (1,49) = 3.946	0.0526
	Genotype x model x treatment	F (1,49) = 0.01559	0.9011
[^{35}S]GTPγS Binding: CA1 (Table 1)	Genotype	F (1,42) = 6.942	< 0.05
	Model	F (1,42) = 3.465	0.0697
	Treatment	F (1,42) = 38.07	< 0.001
	Genotype x model	F (1,42) = 0.3839	0.5389
	Genotype x treatment	F (1,42) = 3.860	0.0561
	Model x treatment	F (1,42) = 0.002648	0.9592
	Genotype x model x treatment	F (1,42) = 3.345	0.0745
[^{35}S]GTPγS Binding: CA3 (Table 1)	Genotype	F (1,43) = 1.272	0.2656
	Model	F (1,43) = 0.1884	0.6664
	Treatment	F (1,43) = 0.4618	0.5004
	Genotype x model	F (1,43) = 0.9209	0.3426
	Genotype x treatment	F (1,43) = 2.187	0.1465
	Model x treatment	F (1,43) = 0.7414	0.3940
	Genotype x model x treatment	F (1,43) = 0.6122	0.4382
[^{35}S]GTPγS Binding: DG (Table 1)	Genotype	F (1,43) = 27.27	< 0.001
	Model	F (1,43) = 1.560	0.2184
	Treatment	F (1,43) = 3.746	0.0595
	Genotype x model	F (1,43) = 1.630	0.2086
	Genotype x treatment	F (1,43) = 22.67	< 0.001
	Model x treatment	F (1,43) = 3.476	0.0691
	Genotype x model x treatment	F (1,43) = 6.050	< 0.05

Measurement	Three-way ANOVA	F(DFn, DFd)	*p*
BDNF CA1 (Table 2)	Genotype	F (1,53) = 1.367	0.2479
	Model	F (1,53) = 40.78	< 0.001
	Treatment	F (1,53) = 1.892	0.1748
	Genotype x model	F (1,53) = 0.2857	0.5952
	Genotype x treatment	F (1,53) = 0.9878	0.3248
	Model x treatment	F (1,53) = 0.2052	0.6524
	Genotype x model x treatment	F (1,53) = 0.02495	0.8751
BDNF CA3 (Table 2)	Genotype	F (1,61) = 1.272	0.7171
	Model	F (1,61) = 0.1884	0.1942
	Treatment	F (1,61) = 0.4618	0.7684
	Genotype x model	F (1,61) = 0.9209	0.5892
	Genotype x treatment	F (1,61) = 2.187	0.4827
	Model x treatment	F (1,61) = 0.7414	0.3613
	Genotype x model x treatment	F (1,61) = 0.6122	0.8092
BDNF DG (Table 2)	Genotype	F (1,54) = 27.16	< 0.001
	Model	F (1,54) = 11.31	< 0.01
	Treatment	F (1,54) = 11.63	< 0.01
	Genotype x model	F (1,54) = 0.5552	0.4594
	Genotype x treatment	F (1,54) = 19.44	< 0.001
	Model x treatment	F (1,54) = 3.206	0.0790
	Genotype x model x treatment	F (1,54) = 3.935	0.0524
